# Cocrystal Applications in Drug Delivery

**DOI:** 10.3390/pharmaceutics12090834

**Published:** 2020-09-01

**Authors:** Andrea Erxleben

**Affiliations:** 1 School of Chemistry, National University of Ireland, H91TK33 Galway, Ireland; andrea.erxleben@nuigalway.ie; 2 Synthesis and Solid State Pharmaceutical Centre (SSPC), V94T9PX Limerick, Ireland

Over the past two decades, considerable research efforts in academia and industry have gone into pharmaceutical cocrystals [1,2]. As a result, a large number of studies on both fundamental aspects and applications of cocrystallisation have been published, and a few cocrystals are now on the market or in clinical trial phases, e.g., sacubitril-disodium valsartan-water (Entresto^TM^), escitalopram oxalate-oxalic acid (Lexapro^®^), ertuglifozin-L-pyroglutamic acid and tramadol-celecoxib. The FDA defines pharmaceutical cocrystals as “crystalline materials composed of two or more different molecules, typically active pharmaceutical ingredient (API) and cocrystal formers (‘coformers’), in the same crystal lattice” [3]. Cocrystallisation is an attractive strategy to modify and improve the physicochemical properties of an API without making covalent changes to the drug molecule itself. Very often cocrystals are designed to tackle the poor dissolution behaviour and low bioavailability of Biopharmaceutics Classification System (BCS) class II and IV drugs that make up 70% of the drug candidates in the development pipeline [4]. However, chemical stability, hygroscopicity, mechanical, and flow properties have also been improved through cocrystal formation [1,2]. Furthermore, cocrystallisation can be used as a purification and enantiomeric separation method [5]. 

This Special Issue includes eight original research articles that highlight the relevance and versatility of pharmaceutical cocrystals in drug delivery.

Machado Cruz et al. report a new cocrystal of the poorly water-soluble antifungal agent itraconazole [6]. They carried out a comprehensive study of the solid-state properties and the formation of the itraconazole-terephthalic acid cocrystal. The cocrystal is stable in aqueous solution and comparison with previously described itraconazole cocrystals showed a correlation of the intrinsic and powder dissolution rates with the solubility of the coformer. The dissolution behaviour of physical mixtures of the cocrystal and common excipients was also analysed. 

Nugrahani et al. prepared the mono- and tetrahydrate of the salt cocrystal diclofenac sodium-l-proline [7]. The hydrates were characterised by single crystal X-ray analysis and were shown to have higher solubilities and dissolution rates than the sodium salt of diclofenac acid and the anhydrous diclofenac acid-l-proline cocrystal. The release of water on drying led to the dissociation of the salt cocrystal into a physical mixture of diclofenac acid and L-proline. Interestingly, this process was reversible. When the dried sample was kept at 72% relative humidity and 25 °C, diclofenac sodium-L-proline tetrahydrate was restored.

A paper by Buol et al. describes the first cocrystals of the nootropic drug nefiracetam [8]. A large cocrystal screen with 133 different coformers using liquid-assisted grinding led to the identification of 13 new cocrystals that were characterised by single-crystal X-ray diffraction. The study illustrates how solid-state properties—in this case, the melting point—can be varied over a wide range by changing the coformer. Three cocrystals with biocompatible coformers were subjected to a more comprehensive screen including solvent evaporation, slurrying and cooling crystallisation. The discovery of additional solid-state forms demonstrates the importance of a thorough screening for multiple cocrystal forms. The solubilities and dissolution properties of the new cocrystals were investigated. 

Kale et al. studied the effect of cocrystallisation on the tabletability of rivaroxaban and found an improved tabletability for rivaoxaban-malonic acid [9]. The tabletability order malonic acid < rivaboxaban < rivaboxaban-malonic acid could be rationalised with the crystal packing, specifically the absence or presence of slip planes, slip plan topology, the degree of intermolecular interactions and d-spacing. Rivaroxaban contains slip planes with a flat-layered topology and with a zigzag layer topology. The crystal structure-mechanical property relationships found in this study shine light on the way crystals that contain more than one slip-plane system deform. 

Wroblewska et al. introduced the new coformer 1-hydroxy-4,5-dimethyl-imidazole 3 oxide [10]. They used high resolution-solid-state NMR to investigate the cocrystal formation with barbituric and thiobarbituric acid during ball-milling. The structures of the new coformer and cocrystals were studied by ^13^C CP/MAS, ^15^N CP/MAS and ^1^H Very Fast MAS NMR in combination with single-crystal X-ray analysis. The effect of the polymorphic and tautomeric form of barbituric/thiobarbituric acid on the cocrystallisation was evaluated. The cocrystals showed no cytotoxicity in HeLa and 293T cells at concentrations of up to 100 µM indicating good biocompatibility of 1-hydroxy-4,5-dimethyl-imidazole 3 oxide. However, the coformer failed to give a significant increase in solubility.

Salas-Zuniga et al. studied the effect of hydroxypropyl methylcellulose and methylcellulose on the dissolution behaviour of two nitazoxanide cocrystals [11]. Using polymer-based powder formulations of nitazoxanide-succinic acid, they achieved a significant improvement of the apparent solubility of nitazoxanide compared to formulations of the pure API. It was suggested that the solubility enhancement was due to a polymer-induced delay of nucleation and crystal growth. 

Witika et al. report a cocrystal of lamivudine and zidovudine [12]. The dual-drug cocrystal was characterised by X-ray powder diffraction, Raman spectroscopy, FT-IR spectroscopy, differential scanning calorimetry and energy-dispersive X-ray spectroscopy. Surfactants were applied to produce and stabilise nano-cocrystals with specific, pre-defined critical quality attributes such as particle size, polydispersity index, and zeta potential to exploit the advantages of nano-sized drug delivery systems. In a follow-up paper, the same authors describe a Design of Experiment approach to optimise the cold sonochemical synthesis of the lamivudine-zidovudine nano-cocrystals in the presence of surfactants and polymers [13]. The nano-cocrystals proved to be less cytotoxic in HeLa cells than a physical mixture of the two APIs.
